# In-home physical frailty monitoring: relevance with respect to clinical tests

**DOI:** 10.1186/s12877-019-1048-8

**Published:** 2019-02-04

**Authors:** Aly Chkeir, Jean-Luc Novella, Moustapha Dramé, Delphine Bera, Michèle Collart, Jacques Duchêne

**Affiliations:** 1 Institut Charles Delaunay, UMR CNRS 6281, Université de Technologie de Troyes (UTT), 12 Rue Marie Curie, CS 42060, F-10004 Troyes, France; 2 Service de Gériatrie, CHU de Reims, Hôpital Maison Blanche, 45 rue Cognacq Jay, F-51092 Reims cedex, France; 3 Pôle recherche et Santé Publique, CHU de Reims, Hôpital Robert Debré, Avenue du Général Koenig, F-51092 Reims, France; 4 Centre Les Arcades, 6 Rue du Pont Royal, F-10000 Troyes, France; 5 Pôle clinique médicale, Centre hospitalier de Troyes, 101 avenue Anatole France, CS 20718, F-10003 Troyes, France

**Keywords:** Frailty, Remote monitoring, Older people

## Abstract

**Background:**

Frailty detection and remote monitoring are of major importance for slowing down, and/or even stopping the frailty process in home-dwelling older people. Taking the Fried’s criteria as a reference, this work aims to compare the results produced by a technological set (ARPEGE Pack) with those obtained by usual clinical tests, as well as to discuss the ability of the Pack to be used for long-run frailty remote monitoring.

**Methods:**

194 participants were given a number of geriatric tests and asked to make use of the ARPEGE technological tools as well as reference clinical tools to feed Fried’s indicators. Spearman or Pearson’s correlation coefficients were used to compare the ARPEGE results to the reference ones, depending on data statistical characteristics.

**Results:**

Good correlations were obtained for measurements of weight (0.99), grip strength (0.89) and walking speed (0.79). Results are much less satisfactory for evaluation of physical activity and exhaustion (Spearman correlation coefficients 0.25 and 0.41, respectively).

**Conclusion:**

Correlations regarding weight, grip strength and walking speed confirm the validity of the data produced by the ARPEGE Pack to feed Fried’s criteria. Assessing activity level and exhaustion from an abbreviated questionnaire is still questionable. However, for long-run monitoring other methods of evaluation can be explored. Beyond the quantitative results, the ARPEGE Pack has been proved to be acceptable and motivating in such a long-term frailty monitoring.

## Background

Population demographics across a large number of countries in Europe and around the world are rapidly changing due to an ageing population. For example, the percentage of the population aged over 65 years in Europe is currently estimated at 18%, a figure that is expected to rise to nearly 30% by the year 2050 [1]. The percentage of the population over 80 years will rise from 4 to 11% over the same period [1]. This increase in the ageing population will have a significant economic impact, due to the healthcare cost for the very old population [2]. This is especially the case when older people gradually become sensitive to stress situations like occurrence of pathology, financial income reduction, break-up of social links etc. They are in a frailty state when they are unable to react to those situations and gradually decline towards dependence if the frailty state is not detected early enough. As a consequence, frail people are unable to achieve the usual activities of daily living. This leads to an inability to live at home without supervision/independently, hence and the need to move to a nursing home.

Detecting the onset of the frailty process early enough in home-dwelling people is of major importance to slow down or even stop the frailty process by an adapted prescription that could be an increase in physical activity, recommendations towards a better diet or a modification in drug delivery. In addition, continuous remote prescriptions follow-up could be useful to measure their effects on a variety of frailty indices and possibly modify them to improve their efficiency [3–8]. Such monitoring can be achieved using in-home technologies that are able to collect, to process and to transmit data from home-dwelling older people provided that those technologies are easily usable, socially acceptable and motivating. It has been shown (see for instance [9]) that older people accept such in-home technologies if the recipients find an improvement in, amongst others, activity and autonomy and if their privacy is respected.

Therefore, technologies for frailty detection and remote monitoring have to be designed with respect to these acceptability conditions and to produce data on the basis of reference scales that can be supplied in a quantified-self manner, or at least with the assistance of a home help or auxiliary nurses. Many of these scales have been published up. The Rockwood and Mitniski [10] model is probably the most comprehensive example (70 items), however it is difficult to apply outside a hospital environment and clearly unsuitable to self-measurement at home. Other scales (see for instance [11, 12]) cover several aspects of frailty (physical, nutritional, emotional …) while being more limited than Rockwood’s in the number of items, but still difficult to adapt to a home context.

Fried et al. [13] described frailty in older adults as a phenotype that could be identified through five criteria: unintentional weight loss, self-reported exhaustion, weakness, slow walking speed, and low physical activity. These criteria have been identified as predictive of health degradation in a number of situations related, for instance, to ageing, cardiovascular problems or renal insufficiency [14–16]. In that way, it would be of great interest to provide general practitioners with data collected from Fried’s scale in a continuous manner. Beyond the fact that this scale has been widely diffused and used to assess physical frailty, scoring its items is easy to perform by means of simple technological tools, hence well adapted to a quantified-self approach. In that way we developed a technological package (the ARPEGE Pack [17]) whose objective was to associate each Fried’s criterion with a wireless device which calculated the criterion value and sent it to a local receiver (typically a tablet or a smartphone) with remote communication capability.

The performance of each technological device included in the ARPEGE Pack was evaluated in a laboratory manner using reference measurements and/or involving only healthy subjects [18–20]. For those reasons, a validation in controlled clinical conditions with older people classified as healthy, pre-frail or frail was necessary in order to show the capability of the ARPEGE Pack to produce a proper estimation of the Fried’s criteria when older people are concerned. The objective of the present work is to compare the results produced by the ARPEGE Pack with those obtained by the usual clinical tests on an ageing population and discuss the ability of that Pack to be used for long-term frailty remote monitoring.

## Methods

### Material

The ARPEGE Pack aims to provide the data for the five Fried’s criteria by means of technological devices. The weight (then weight loss after successive measurements) is obtained by a connected bathroom scale which, in addition, includes a functionality evaluating balance quality (BQT: Balance Quality Tester [21]). In Fried’s work, weakness is evaluated by means of grip strength measurements using a Jamar hand-held dynamometer. In order to make it easy and motivating to use in long-term monitoring, we developed the “Grip-ball” [19], a ball including all functionalities for measuring and wirelessly communicating the pressure exerted on it by the subject. The Grip-ball can be associated with a serious game which ensures the motivation side of the device [22]. As clinicians generally refer to force instead of pressure, a regression model has been developed to convert pressure into force [23]. In the ARPEGE study, we made use of an intermediate version of the Grip-ball where electronic and communication hardware was implemented outside the ball. Concerning walking speed, its evaluation is typically achieved in clinical practice by timing the subject walking over a specific distance (15 ft in Fried’s work). The device included in the ARPEGE Pack makes use of a Doppler sensor (X-Band Doppler Motion Detector MDU 1130, Microwave Solutions Ltd., Marlowes, UK) associated with hardware for conditioning and communication needs, hidden in an object usually encountered at home (a vase in the current ARPEGE demonstrator) [20]. The two remaining Fried’s criteria are assessed in the ARPEGE Pack by means of questionnaires included in the local receiver (a tablet in the present study). We had neither the time nor the wish to use the Minnesota Leisure Time Activity questionnaire extensively [24] for assessing physical activity level during the experiment, even in its short form. We preferred to reduce that assessment to one question adapted in French from that used in the SHARE Project (“How often do you engage in activities that require a low or moderate level of energy such as gardening, cleaning the car, or going for a walk?” [25]). For exhaustion evaluation, we adapted in French the two questions used by Fried et al. [13] and derived from the CES-D scale [26].

### Protocol

One hundred ninety-four participants (Table 1) were recruited from three different locations: 141 patients coming for a geriatric examination at the Reims University Hospital (CHU Reims, France) and the Troyes non-University Hospital (CH Troyes, France), and 53 healthy older people participating in social activities organized by the Arcades First-line Prevention Center in Troyes (France). Inclusion lasted from 2013 to 10-01 to 2014-09-30. It was limited to an age of 70 years and over. People unable to stand up, with severe handicaps, acute pathologies or severe cognitive disorders (Mini-Mental State Examination < 10) were excluded from the experiment.

**Table 1:** Population characteristics

	Men (*n* = 78)		Women (*n* = 116)		mixt (*n* = 194)	
	Mean	SD	Mean	SD	Mean	SD
Age (years)	78.2	±5.2	79.4	±6.0	78.9	±5.7
Height (cm)	170.4	±6.7	156.8	±6.1	162.2	±9.2
Activities of Daily Living (ADL)	5.8	±0.4	5.7	±0.4	5.8	±0.4
Instrumental Activities of Daily Living (IADL)	5.8	±2.1	6.8	±1.8	6.4	±2.0
Balance disorders (Berg’s scale)	52.1	±4.2	50.4	±6.4	51.1	±5.7
Timed Up and Go test (TUG (s))	11.7	±9.0	13.1	±8.0	12.5	±8.4
Mini-nutritional Assessment – Short form (MNA-SF)	9.4	±1.9	9.0	±2.0	9.1	±1.9
Risk of developing pressure sores (NORTON’s scale)	19.5	±1.1	19.3	±1.1	19.4	±1.1
Mini-geriatric depression scale (MINI_GDS)	0.4	±0.8	0.9	±1.1	0.7	±1.0
Duke Health Profile (DUKE’s scale)	21.4	±4.4	20.1	±4.4	20.6	±4.5
Comorbidity index (CHARLSON’s scale)	0.9	±1.1	0.6	±0.8	0.7	±0.9
Weight (kg)	80.0	±12.0	65.9	±12.6	71.6	±14.2
Walking speed (m/s)	0.78	±0.5	0.74	±0.4	0.75	±0.4
Grip Strength (Jamar (kg))	42.6	±18.9	25.6	±11.3	32.4	±17.0
Time to walk 15 ft (s)	4.8	±1.4	6.1	±3.5	5.6	±2.9

Table show both the mean and the standard deviation of the main items evaluated

After providing socio-demographic data, participants underwent Comprehensive Geriatric Assessment, including level of dependency (using Katz activities of daily living (ADL) [27], and Lawton & Brody’s instrumental activities of daily living [28]), balance disorders (using Berg’s scale [29], walking difficulties (using the timed get-up and go test [30], nutritional status (using the Mini-nutritional Assessment – Short form [31], risk of developing pressure sores (using the Norton scale [32]), risk of depression (using the mini-geriatric depression scale [33]), Health-related quality of life (using the Duke Health Profile [34]), and the level of comorbidity (using the Charlson’s comorbidity index [35]). In a second step subjects were asked to declare their current weight, test their maximal grip strength using the reference device, i.e. the Jamar dynamometer [36], as well as to walk 15 ft at their usual pace, the measure being the time to cross the distance. All data described above were first captured manually in a paper document and then electronically recorded on the Reims University server for further processing. Subjects were also asked to use the ARPEGE Pack to measure their weight (stepping on the bathroom scales), maximal grip strength (using the Grip-ball prototype), and walking speed (walking 15 ft at usual pace in the radar direction), then to answer the questions related to activity level and exhaustion (multiple choice on the tablet). All tests were achieved under the supervision of a clinical investigator. Sufficient periods of rest were preserved between successive tests involving a physical activity.

### Data processing

As the objective is to compare the criterion values produced by clinical evaluation and by the ARPEGE Pack, a linear regression model was calculated between quantitative data under the condition that they were Gaussian. Normality was verified for weight, grip strength and walking speed using the Kolmogorov-Smirnov statistical test (threshold *p* = 0.05). The scores produced by the questionnaires being obviously non-Gaussian, the comparison between clinical tests and ARPEGE questionnaires was achieved by means of the Spearman correlation coefficient. The good agreements between the two approaches was assessed using the Cohen’s kappa test. All statistical tests were performed using the XLSTAT statistical package (XLSTAT Version 2015.6.01.23953, Addinsoft, Paris, France) and the Statistical Package for Social Sciences (SPSS Inc., Chicago, IL, USA). *P* values less than 0.05 were considered to be statistically significant.

## Results

### Weight: Bathroom scale measurement vs. declaration

**Fig. 1:**
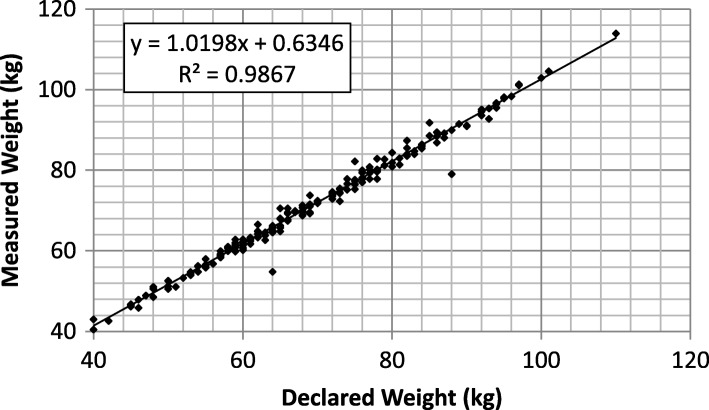
Linear regression between weights declared by the subjects (horizontal axis) and measured by the BQT (vertical axis)

The bathroom scales had been calibrated before starting the experiment session, the only result to be shown is the relationship between the weight as declared by the subject and the value produced by the scale for the same subject (Fig. 1). Both outcomes are obviously highly correlated, with a small intercept value due to the fact that the subjects were not asked to get undressed for the test. In addition, when excluding the two obvious outliers in Fig. 1, the coefficient of determination R^2^ increases to 0.993. These outliers were probably caused by an error in transcription from the paper document to the server.

### Grip-strength: Grip-ball vs. Jamar

As the Grip-ball sensor produces a value of pressure P_T_ corresponding to the sum of the pressure P_A_ exerted by the subject and the atmospheric pressure P_I_, it was necessary to convert the results from pressure to force before comparing Grip-ball data with the reference values produced by the Jamar. The conversion model used in the present study can be found in [19]:

F = (4.855 × 10^‐3^P_I_ ‐ 2.020 × 10^‐2^)P_A_with force expressed in kg and pressure in (kPa)

**Fig. 2:**
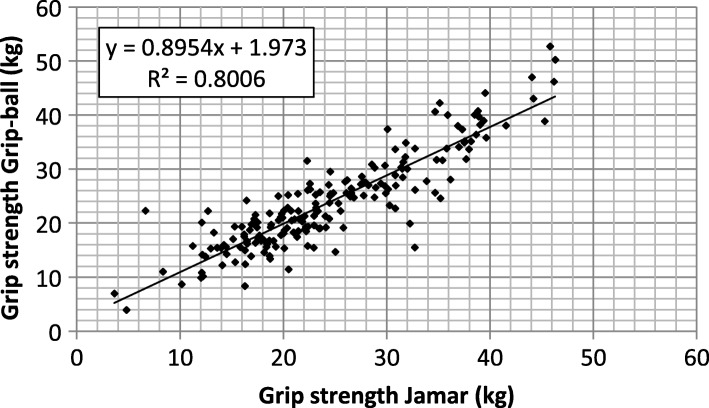
Linear regression between grip forces measured by the JAMAR reference device (horizontal axis) and the Grip-ball (vertical axis)

Results are displayed in Fig. 2. The slope is underestimated (0.89 instead of 1 expected) and the intercept close to 2 kg. The coefficient of determination R^2^ is equal to 0.80, i.e. Pearson correlation coefficient equal to 0.89. If we set intercept to 0, the slope increases to 0.967 without a significant decrease in the coefficient of determination (from 0.800 to 0.795).

### Walking speed: Radar vs. stopwatch

**Fig. 3:**
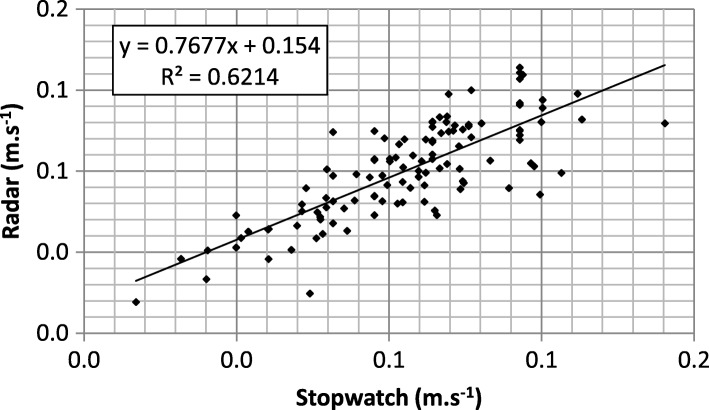
Linear regression between walking speeds obtained by timing the subjects (horizontal axis) and measured by the Radar device (vertical axis)

Figure 3 shows the relationship between values produced by the Radar device and those obtained by timing subjects over the distance advocated by Fried et al. (15 ft). Notice that the two experiments were not achieved simultaneously, so that subjects were asked to walk two times with the same instruction of “at usual pace”. When forcing the intercept to 0, the slope becomes 0.94 and the coefficient of determination decreases from 0.62 to 0.59, i.e. Pearson correlation coefficients of 0.79 and 0.77, respectively.

### Exhaustion and activity level: ARPEGE vs. partial score of clinical questionnaires

As we did not have a Gold standard to measure activity level and exhaustion, we extracted the results produced by the few items more or less related either to activity or to a depression state from Duke Health Profile and mini-GDS.

#### Activity level compared to Duke partial score (items 8, 9 and 16)

We selected 3 items roughly related to a physical activity:

- Item 8. “Today would you have any physical trouble or difficulty when walking up a flight of stairs”
- Item 9. “Today would you have any physical trouble or difficulty when running the length of a football field”
- Item 16. “During the past week, how often did you take part in social, religious, or recreation activities?”

We obtained r = 0.250 for Pearson’s correlation coefficient computed between ARPEGE activity score (one question, see § 2.1) and the sum of Duke items 8, 9 and 16. Even though significant, correlation is very poor, expressing the obvious lack of correspondence between both questionnaires.

#### Exhaustion: ARPEGE questionnaire compared to mini-GDS and Duke partial score (items 4 and 13)

The two selected items from Duke Health Profile are expressed as (before translation to French):

- Item 4. “I give up too easily”
- Item 13. “During the past week: How much trouble have you had with feeling depressed or sad?”

Pearson’s correlation value (r = 0.414) was a little better than those obtained for activity level but remained low if the objective is to replace the one by the other. As a comparison, correlations were also calculated between ARPEGE score and mini-GDS (r = 0.340) and between mini-GDS and the selected items of the Duke Profile (*r* = 0.680).

## Discussion

The ARPEGE Pack has been designed for long-term follow-up of frail people at their usual place of residence. It can be used either by older people themselves in a quantified-self manner or by home care professionals (daily care or home help), depending on the person’s dependency level. It is intended for assessing older people’s frailty level and monitoring the effect of prescriptions on health and well-being. This implies providing older people themselves and healthcare professionals with information related to the trend of the frailty indicators in real time. This means that data produced at a specific day is less of interest than the trend evaluated over a week or even a month. Though individual measurements are locally displayed on the tablet or the Smartphone (except for the walking speed, the measurement of which is totally blind to the user), they are automatically sent to a remote server where they are synthetized, typically into a moving average on one week, and displayed as trend curves on a Web site accessible by login and password to the users themselves and possibly the clinician and/or general practitioner. Health professionals are especially involved when monitoring has been proposed to follow the improvement in a person’s health state further to a medical prescription (adapted physical activity, diet recommendations, modification in drug prescription etc.).

All devices included in the ARPEGE Pack have been designed to make them acceptable (even attractive) for long-term monitoring. No one would be surprised to see scales in a bathroom, and weight measurement made by the BQT does not need any specific protocol except the need to wait for the weight to be displayed on the scale screen before getting down off the scales. Communication between the scales and the local receiver (tablet or Smartphone) does not need any action from the user. On the other hand, the Grip-ball requires the user to manipulate it in order to produce a series of grip strength values day after day. Such a requirement could be demotivating over the long term if there is no attractive way to practice. This is the reason why we suggest associating the Grip-ball with a serious game [22], the score being transmitted to the local receiver only when the player identifies as the monitored person.

Relevance of the grip strength value produced by the Grip-ball has to be evaluated from Fig. 2. From the coefficient of determination (0.80), the corresponding Pearson’s correlation coefficient (0.89) is equivalent to the result of Desrosiers et al. [37] who found a Pearson’s correlation coefficient of 0.89 or 0.90 depending on the hand used.

**Fig. 4:**
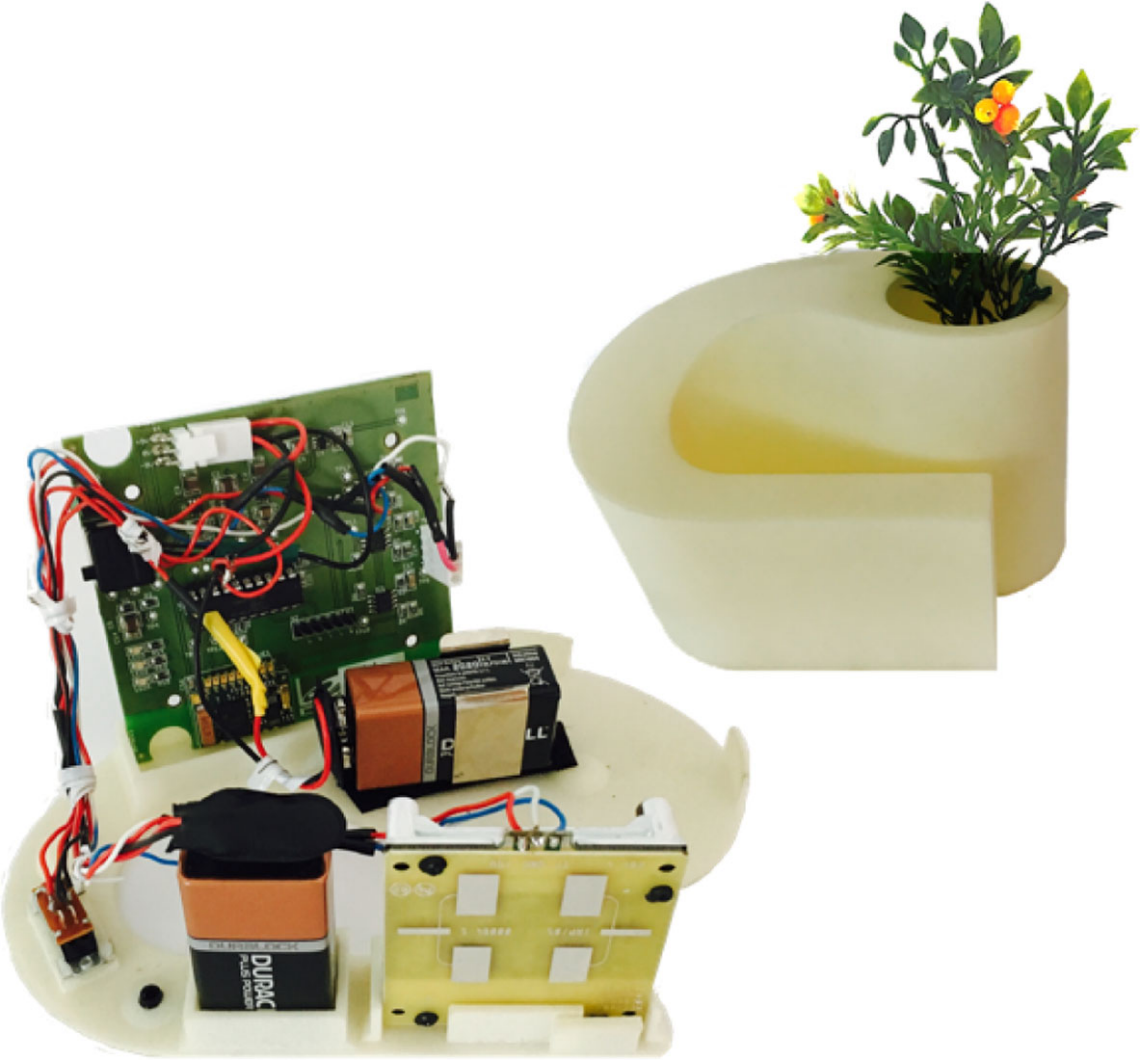
Prototype of the Radar device and its inclusion in a vase

The Radar device has to be put in a location where it is possible to measure a walking speed on a distance of 2.5 to 3 m (the maximum distance to ensure a proper detection by the currently available device). Such a distance can be usually found in most homes, even though we must recognize that it could be a limitation. Social acceptation of the device has been taken into account by including sensor and associated electronics in an object currently encountered at home (specifically a vase, Fig. 4). In its first version, the prototype was designed to be permanently activated. We are about to upgrade to a second version, including a presence detector, in order to produce and communicate a walking speed value to the local receiver only if there is someone walking in its direction. In addition tests are scheduled in Living Lab in order to determine how to select only the values deduced from older people’s genuine walk. This is achievable due to the fact that the sensor produces an instantaneous speed value. Therefore it should be possible to classify walk and non walk movements by analysing the signal profile produced by the sensor.

In terms of speed values, comparison between Radar and stopwatch measurements leads to a coefficient of determination of 0.62, decreasing to 0.59 when forcing the intercept to 0. Hence a Pearson’s correlation coefficient of 0.79 and 0.77, respectively. Even though these results sound satisfactory, discrepancies between both measurement methods would have two main origins. The first is investigator dependent timing: defining precisely the crossing of start and stop lines manually could lead to differences in results from one trial to another, especially for high speeds, i.e. low timing values. The second relates to the intra-subject variability when achieving the two trials separately, which was the case in the present work. When asking subjects to walk at a comfortable gait speed, Bohannon [38] found a high correlation (0.903) between two successive trials. However, the people included were healthy individuals between 20 and 79 years of age whose physical shape and capacity were quite different from those of our population. In a previous work [20], we evaluated in the same way (Radar and stopwatch) the walking speed of twenty three young and healthy subjects, asked to walk 10 times down a corridor over a maximum distance of 10 m at three self-determined speeds (slow, usual, fast). The resulting correlation between Radar and stopwatch measurements was around 0.90, slightly depending on the trial conditions. This favors an assumption of higher test-to-test variability for older people.

*Cohen’s kappa coefficient was calculated to determine the agreement between the two approaches, with (k) = 0.7613; (k) showed good agreements between the two approaches (ARPEGE* VS. *FRIED’s SCALE). The number of subjects classified as frail and non-frail for each of the two approaches is shown in the* Table 2*.*

**Table 2:** The number of subjects classified as frail and non-frail for each of the two approaches is shown in this table

Technological tools		Arpege	
		Frail	Not frail
Fried’s scale	Frail	5	1
	Not frail	2	186

Results related to physical activity and exhaustion sound the more disappointing. However, they need to be carefully interpreted due to the fact that the clinical items used as references do not correspond to a “Gold Standard” with respect to those included in the ARPEGE Pack. On one hand, exhaustion assessment was made by a French adaptation of the two questions used by Fried et al. [13] and derived from the CES-D scale [26, 39]. We should point out that CES-D does not reflect what could be produced by the mini-GDS clinical test. On the other hand, we did not (and did not want to) dispose of a relevant but time-consuming clinical tool like the Minnesota LTA questionnaire dedicated to physical activity assessment. This obviously constitutes a handicap in our attempt to evaluate the relevance of the SHARE single question related to physical activity. Therefore the results obtained when comparing answers produced either by the SHARE question or a Duke partial score are far from being satisfactory, given that we did not compare equivalent questionnaires. However, putting the work in context, the ARPEGE Pack’s main purpose is a long-run monitoring of frailty evolution over time. In that case the ARPEGE Pack could include some activity sensors [40, 41] such as those commercially on the market or even included in Smartphones.

Finally, we propose two hypotheses to explain why we have only 8 people classified as frail (Table 2). The first hypothesis concern the effects of subjects’ age, 194 participants were recruited from three different locations as it presented in the protocol section. It was limited to an age of 70 years and over, the age of subjects are distributed in percentage as: (66% of subjects are aged < 80 years; 80 years < 34% of subjects are aged < 90 years). In this study, we noticed that six subjects among the eight classified frail, are aged greater than 80 years. Authors take this result into account before any new future recruitment could take place, for example: we can reverse the recruitment process (i.e. 66% > 80 years; 34% < 80 years). The second hypothesis more probable than the first, in our opinion, this might be due to the strict cut-offs used in Fried conditions, especially for grip-strength and walking speed. In fact, the Fried cut-offs, were based on US community data from the CHS (Cardiovascular Health Study). In addition, it may be useful to highlight what we observed about the difference in the average heights for example. There was a gap of about 2.6 cm for men and 2.4 cm for women between our subjects and Fried’s group. By adjusting the Fried cut-off for these two new values, we observed that 7.27% of our subjects passed from Not-frail to Frail. Hence, it may be preferable to associate data from several European geriatric centers in order to develop particular cut-offs for European populations.

## Conclusion

Remote monitoring of physical the frailty state and its evolution is part of the great challenge aiming at allowing older people to live at home while preserving their autonomy and well-being. There are two main conditions that have to be followed by such a monitoring system in order to be efficient over time. The first condition is the relevance of the data produced by the sensors with respect to reference clinical tests. The ARPEGE Pack roughly fits this condition, especially for the Fried’s indicators objectively measured by technological tools (weight, grip strength, walking speed). The second condition relates to acceptability and motivation. When people are requested to achieve a specific action day by day, this action must be either designed to provide pleasure in doing it, which is the case for the Grip-ball associated with a serious game, or a normal part of everyday life, like using the bathroom scales. When sensors do not require any voluntary action from the user (case of the Radar device) they have to be hidden in socially acceptable objects, i.e. usually encountered at home like the vase proposed in the ARPEGE Pack.

The next step will be to test the ARPEGE Pack in real conditions at home. Such an experiment is currently being set up in association with a teleservice platform and with the active participation of the general practitioners who usually follow the patients included in the experiment.

## Abbreviations

ADL: Activities of Daily Living
ARPEGE: Anticipation et Repérage: Pack d’évaluation Gérontologique Embarqué
BQT: Balance Quality Tester
CES-D: Center for Epidemiologic Studies- Depression
CHS: Cardiovascular Health Study
IADL: Instrumental Activities of Daily Living
TUG: Timed Up and Go test

## References

[1] DGEFA E: The 2005 EPC projections of age-related expenditure (2004–50) for the EU-25 member states. In: *European Economy.* Edited by affairs ECD-GfEaF; 2005.

[2] Martins JO, De la Maisonneuve C, Bjornerud S: Projections of OECD health and long-term care public expenditures. In: Banca d’Italia public research department public research workshop, fiscal indicators; Banca d’Italia: Perugia, Italy. 2006: 753–793.

[3] Gustafsson S, Edberg A-K, Johansson B, Dahlin-Ivanoff S (2009). Multi-component health promotion and disease prevention for community-dwelling frail elderly persons: a systematic review. Eur J Ageing.

[4] Giné-Garriga M, Roqué-Fíguls M, Coll-Planas L, Sitjà-Rabert M, Salvà A (2014). Physical exercise interventions for improving performance-based measures of physical function in community-dwelling, frail older adults: a systematic review and meta-analysis. Arch Phys Med Rehab.

[5] Cadore EL, Rodriguez-Manas L, Sinclair A, Izquierdo M (2013). Effects of different exercise interventions on risk of falls, gait ability, and balance in physically frail older adults: a systematic review. Rejuvenation Res.

[6] Pialoux T, Goyard J, Lesourd B (2012). Screening tools for frailty in primary health care: a systematic review. Geriatr Gerontol Int.

[7] Elkan R, Kendrick D, Dewey M, Hewitt M, Robinson J, Blair M, Williams D, Brummell K (2001). Effectiveness of home based support for older people: systematic review and meta-analysis. BMJ.

[8] Bauer M, Fitzgerald L, Haesler E, Manfrin M (2009). Hospital discharge planning for frail older people and their family. Are we delivering best practice? A review of the evidence. J Clin Nurs.

[9] Morris ME, Adair B, Miller K, Ozanne E, Hansen R, Pearce AJ, Santamaria N, Viegas L, Long M, Said CM (2013). Smart-home technologies to assist older people to live well at home. Aging Sci.

[10] Rockwood K, Mitnitski A (2007). Frailty in relation to the accumulation of deficits. J Gerontol A Biol Sci Med Sci.

[11] Strawbridge WJ, Shema SJ, Balfour JL, Higby HR, Kaplan GA (1998). Antecedents of frailty over three decades in an older cohort. J Gerontol B Psychol Sci Soc Sci.

[12] Studenski S, Hayes RP, Leibowitz RQ, Bode R, Lavery L, Walston J, Duncan P, Perera S (2004). Clinical global impression of change in physical frailty: development of a measure based on clinical judgment. J Am Geriatr Soc.

[13] Fried LP, Tangen CM, Walston J, Newman AB, Hirsch C, Gottdiener J, Seeman T, Tracy R, Kop WJ, Burke G (2001). Frailty in older adults: evidence for a phenotype. J Gerontol A Biol Sci Med Sci.

[14] Singh M, Stewart R, White H (2014). Importance of frailty in patients with cardiovascular disease. Eur Heart J.

[15] Sinclair M, Poltavskiy E, Dodge J, Lai J (2017). Frailty is independently associated with increased hospitalisation days in patients on the liver transplant waitlist. World J Gastroenterol.

[16] McAdams-DeMarco M, Isaacs K, Darko L, Salter M, Gupta N, King E, Walston J, Segev D (2015). Changes in frailty after kidney transplantation. J Am Geriatr Soc.

[17] Jaber R, Chkeir A, Hewson D, Duchêne J: ARPEGE: Assessment of Frailty at Home. In: *Healthcom* 2014*: 2013; Lisbon*.

[18] Hewson D, Duchêne J, Hogrel J-Y (2015). Validation of balance-quality assessment using a modified bathroom scale. Physiol Meas.

[19] Chkeir A, Jaber R, Hewson D, Duchêne J (2013). Estimation of grip force using the grip-ball dynamometer. Med Eng Phys.

[20] Jaber R, Chkeir A, Hewson D, Duchêne J: A new device to assess gait velocity at home. In: *Medicon; Sevilla* 2013.

[21] Duchêne J, Hewson D (2011). Longitudinal evaluation of balance quality using a modified bathroom scale: usability and acceptability. J Telemedicine Telecare.

[22] Chkeir A, Voilmy D, Duchêne J, Hewson DJ: Does the use of a serious game and the grip-ball decrease discomfort in older people when assessing maximal grip-strength? In: *Medicon* 2016*: March 31–April 2nd; Paphos, Cyprus*. 2016.

[23] Jaber R, Hewson DJ, Duchêne J (2012). Design and validation of the grip-ball for measurement of hand grip strength. Med Eng Phys.

[24] Taylor HL, Jacobs DR, Schucker B, Knudsen J, Leon AS, Debacker G (1978). A questionnaire for the assessment of leisure time physical activities. J Chronic Dis.

[25] Romero-Ortuno R, Walsch CD, Lawlor BA, Kenny RA (2010). A frailty instrument for primary care: findings from the survey of health, ageing and retirement in Europe (SHARE). BMC Geriatr.

[26] Radloff LS (1977). The CES-D scale: a self-report depression scale for research in the general population. Appl Psychol Meas.

[27] Katz S, Ford A, Moskowitz R, al. E: the index of ADL: a standardized measure of biological and psychosocial function. JAMA 1963, 185(12):914–919. 10.1001/jama.1963.03060120024016 14044222

[28] Lawton M, Brody E (1969). Assessment of older people: self-maintaining and instrumental activities of daily living. The Gerontologist.

[29] Berg K, Norman KE (1996). Functional assessment of balance and gait. Clin Geriatr Med.

[30] Podsiadlo D, Richardson S (1991). The timed "up & go": a test of basic functional mobility for frail elderly persons. J Am Geriatr Soc.

[31] Kaiser M, Bauer J, Ramsch C, Uter W, Guigoz Y, Cederholm T, Thomas D, Anthony P, Charlton K, Maggio M (2009). Validation of the mini nutritional assessment short-form (MNA-SF): a practical tool for identification of nutritional status. J Nutr Health Aging.

[32] Norton D, McLaren R, Exton-Smith AN: An Investigation Of Geriatr Nurs Problems In Hospital, vol. 77. London; 1962.

[33] Clément J, Preux PM, Fontanier D, JM. L: mini-GDS in elderly population administered by general practitioners. Encephale 2001, 27(4):329–337. 11686054

[34] Parkerson GRJ, Broadhead WE, Tse CK (1990). The Duke health profile. A 17-item measure of health and dysfunction. Med Care.

[35] Charlson M, Szatrowski TP, Peterson J, Gold J (1994). Validation of a combined comorbidity index. J Clin Epidemiol.

[36] Schmidt RT, Toews JV (1970). Grip strength as measured by the Jamar dynamometer. Arch Phys Med Rehab.

[37] Desrosiers J, Hebert R, Bravo G, Dutil E (1995). Comparison of the Jamar dynamometer and the Martin vigorimeter for grip strength measurements in a healthy elderly population. Scand J Rehabil Med.

[38] Bohannon RW (1997). Comfortable and maximum walking speed of adults aged 20-79 years: reference values and determinants. Age Ageing.

[39] Orme J, Reis J, Herz E (1986). Factorial and discriminate validity of the Center for Epidemiological Studies depression (CES-D) scale. J Clin Psychol.

[40] Yang CC, Hsu YL (2010). A review of Accelerometry-based wearable motion detectors for physical activity monitoring. Sensors (Basel).

[41] Schwenk M, Mohler J, Wendel C, D'Huyvetter K, Fain M, Taylor-Piliae R, Najafi B (2015). Wearable sensor-based in-home assessment of gait, balance, and physical activity for discrimination of frailty status: baseline results of the Arizona frailty cohort study. Gerontology.

